# Congenital Brucellosis: A Case Report and Literature Review

**DOI:** 10.7759/cureus.79736

**Published:** 2025-02-27

**Authors:** Eman J Ghazwani, Mohaned Mohammed, Ahmed Albishri, Hadeel Almehery, Samah E Mohammed, Shady Wafa

**Affiliations:** 1 Pediatric Infectious Diseases, Armed Forces Hospital Southern Region, Khamis Mushait, SAU; 2 Pediatric Medicine, Armed Forces Hospital Southern Region, Khamis Mushait, SAU; 3 Pediatrics, Armed Forces Hospital Southern Region, Khamis Mushait, SAU

**Keywords:** brucella, congenital brucellosis, neonate, pregnancy, saudi arabia

## Abstract

Brucellosis, the most common bacterial zoonotic infection in many countries, is endemic to the Kingdom of Saudi Arabia. Brucellosis develops due to infection by the Gram-negative genus *Brucella*. Brucellosis is usually transmitted by the consumption of infected animal milk or dairy products and is rarely transmitted through human-to-human transmission. However, cases of transmission have been documented, resulting from blood transfusions, transplantation, breastfeeding, sexual contact, and aerosol transmission. Affected neonates may manifest with low birth weight, jaundice, hepatosplenomegaly, respiratory distress, and sepsis, but some cases are asymptomatic. Herein, we present the case of a severely preterm neonate who presented with severe respiratory distress and sepsis after delivery. The neonate was positive for Gram-negative coccobacilli, and the mother had a history of contact with animals during pregnancy, leading to a high suspicion for and a diagnosis of congenital brucellosis. Brucellosis should be closely monitored, especially in epidemic areas. Congenital brucellosis treatment in newborns faces additional challenges of drug choice and disease relapse. We treated our patient with trimethoprim/sulfamethoxazole and rifampin for six weeks, and she was discharged in good condition.

## Introduction

Brucellosis is considered one of the most common zoonotic infections in the Kingdom of Saudi Arabia (KSA). It is caused by a Gram-negative, non-encapsulated, and non-motile coccobacillus, which has many species, including Brucella (B.) melitensis, B. abortus, B. suis, and B. canis, in which sheep, cattle, swine, and dogs are reservoirs, respectively. In 2011, the Saudi Ministry of Health reported an incidence of 18/100,000 population per year, with seroprevalence rates of 15% in the general Saudi population, and 10% in children 0-14 years of age [1]. Congenital brucellosis affects 2% of newborns exposed to brucellosis in utero, during fetal life [2]. Brucellosis is usually transmitted to humans via direct contact with infected animals or the consumption of infected products such as meat or unpasteurized milk [1]. Human-to-human transmission is rare but vertical transmission from mother to infant through breast milk has been reported [3]. Brucellosis also can be transmitted by other mechanisms such as bone marrow transplantation [4], sexual intercourse [5], and blood transfusion [6]. Congenital brucellosis is extremely rare in the KSA but can be transmitted across the placenta in mothers with bacteremia or through contact with maternal blood, urine, or genital secretion during childbirth [7]. Infected newborns may have low birth weight, jaundice, hepatosplenomegaly, respiratory distress, neonatal sepsis with fever, and vomiting, but some cases are asymptomatic.

Herein, we present a preterm neonate who was successfully treated for confirmed congenital Brucellosis.

## Case presentation

A preterm female of 29+4 weeks gestation was delivered to a symptomatic 37-year-old Saudi mother, P5L4A2, with a history of early neonatal death due to severe intrauterine growth restriction. The mother received regular follow-up during pregnancy, during which time she took insulin for type 2 diabetes mellitus. In addition, the mother had no premature rupture of membranes, her antenatal scan was normal, and she was Rhesus factor negative, for which she received anti-D immunoglobulin at 28 weeks of gestation. The mother was admitted due to premature labor, laboratory investigation of the mother revealed a white blood cell count of 26,000, a hemoglobin level of 13.9 g/dL, a platelet count of 148,000, and a C-reactive protein (CRP) of 29.2 mg/L.

The baby was delivered via emergency cesarean section due to premature labor. She was intubated and connected to a portable mechanical ventilator due to severe respiratory distress before being transferred to the neonatal intensive care unit. Upon examination, the baby had a birth weight of 1495 kg, a head circumference of 28 cm, a length of 41 cm (ponderal index was 3.6 and all growth parameters in the 50th centile according to the Saudi growth chart), a heart rate of 156 beats/minute, a respiratory rate of 65 breaths/minute, a temperature of 36.7 ℃, and a blood pressure of 60/40 mmHg. She was not dysmorphic, but tachypneic, with subcostal and intercostal retraction, and decreased air entry bilaterally. She was connected to a mechanical ventilator, synchronized to intermittent positive pressure ventilation mode due to severe respiratory distress, and received surfactant. She had normal heart sounds with no murmur. Her abdomen was soft with no organomegaly. She had normal female genitalia and was hypoactive, with normal tone, power, and reflex with a Ballard score of 28.

The newborn was treated empirically with antibiotics due to suspicion of early-onset sepsis. Blood samples were obtained for cultures. She was started on ampicillin and gentamicin, and blood tests were done (Table 1). Cefotaxime was added due to worsening of the patient’s condition, leukocytosis, and rising CRP, which was stopped after three days when blood culture results showed no growth after 48 hours of incubation.

**Table 1:** Hematological and biochemical investigations tWBC: total white blood cell, Hb: hemoglobin, CRP: C-reactive protein

Laboratory Test	Result	Units	Reference Range
tWBC	35.13	x 10^9^/L	4.5–13.5
Hb	13.9	g/dL	10.9–15
Platelet	211	x10^9^/L	150–450 × 10^9^/L
CRP	29	mg/L	<6
Serum bilirubin	36.3	mmol/L	17.1-205
Brucella microagglutination test (at day 6 of age)	1:80		<1:80

At 12 hours of age, the patient was noted to have jaundice. Phototherapy was started, to which the patient responded well. On day 6 of age, Gram-negative coccobacilli was detected on the first blood culture. Her cerebrospinal fluid (CSF) cell count was normal, CSF culture was negative, viral and brucella CSF polymerase chain reaction was negative, and serum agglutination test was negative with titer <1:80 for Brucella abortus and Brucella melitensis. The pediatric infectious disease unit then modified the antibiotic treatment regimen. Ampicillin was stopped, gentamycin was discontinued for seven days, trimethoprim/sulfamethoxazole was added, plus rifampin orally via nasogastric tube, and the mother was screened for Brucella. Serum agglutination tests were positive for B. melitensis and B. abortus, with titers of 1:640 and 1:320, respectively. Therefore, the mother was started on treatment accordingly.

On day 23 of life, the patient developed abdominal distension and increased abdominal girth and was treated for necrotizing enterocolitis (NEC) stage one. She was kept nil per os. Her oral medication was stopped and changed to IV ciprofloxacin and IV trimethoprim/sulfamethoxazole, plus vancomycin. Her general condition gradually improved, and she started feeding. Her IV medication was stopped on day 28 of life, and she was shifted to oral trimethoprim/sulfamethoxazole and rifampin for a total of six weeks. The patient was discharged on day 50 of life. At discharge, she was fully on oral feeling, in room air, and weighed 2 kg. She received routine childhood immunizations and showed normal development at the age of 12 months in the outpatient clinic.

## Discussion

Brucellosis is one of the most common zoonotic bacterial infections affecting all age groups. In this case, as our patient had not been breastfed or received a blood transfusion, these routes of transmission were unlikely. Therefore, the baby most likely was infected in utero via the placenta. The mother’s infection was supported by her history of frequent visits to her family farm, with her serological findings indicating that intrauterine transmission of brucellosis took place. The mother had minor symptoms during pregnancy, including headache and malaise, and she most likely developed preterm labor as a complication of untreated brucellosis in pregnancy [8]. The mother was diagnosed with brucellosis based on her medical history and serological tests (i.e., serum agglutination test).

In the acute phase of brucellosis, blood cultures have an accuracy rate of only 40%-70%, and negative serological tests should never exclude a diagnosis of the disease in neonates [9]. Early diagnosis with appropriate treatment can improve patient outcomes. Congenital brucellosis should be considered in severely ill newborns, in endemic places, and after the exclusion of other infections [8]. In our case, the diagnosis of brucellosis was confirmed by positive blood culture. Although many antimicrobial agents are active against Brucella spp in vitro, their clinical effectiveness varies. Furthermore, as there is a risk of relapse with monotherapy, combination therapy is generally recommended.

Treatment options for brucellosis depend on the patient’s age. Children >8 years of age are recommended oral doxycycline/rifampin for 6-8 weeks, whereas children <8 years of age should receive oral trimethoprim/ sulphamethoxazole plus rifampin for 6-8 weeks. In our case, before the identification of brucella, the patient was treated empirically with ampicillin/gentamycin and cefotaxime. After her blood culture results back came negative, cefotaxim was discontinued, and ampicillin/gentamycin was planned for seven days. After the diagnosis of brucellosis, her treatment was changed to trimethoprim/sulphamethoxazole plus rifampin. Gentamycin was stopped on day 9 of life because the patient developed NEC. Her regimen was then changed to IV ciprofloxacin, trimethoprim/sulphamethoxazole IV, and vancomycin for five days, and then she resumed her previous medication regimen for a total of six weeks.

Early diagnosis and appropriate treatment are crucial for prognosis, as congenital brucellosis has high rates of mortality and morbidity. Physicians should have a high suspicion of brucellosis for patients living in endemic areas, as no vaccine currently exists for use in children. Public education continues to have a prominent role in the prevention of brucellosis. In our case, the neonate was delivered preterm, showed signs of respiratory distress and possible sepsis, leukocytosis, and high CRP, and her mother had a history of contact with animals in an endemic area.

## Conclusions

A history of travel to endemic regions, as well as consumption of exotic food or unpasteurized dairy products, may be an important clue for diagnosing human brucellosis. Taking a detailed history from parents is very important to reach an early diagnosis and start treatment as soon as possible. Congenital brucellosis should be suspected in neonate cases when other bacterial infections have been excluded, especially if the mother’s history is consistent with Brucella exposure. Treatment of brucellosis in neonates faces the challenges of drug choice and disease relapse. Unfortunately, there is no effective vaccine that can protect against brucellosis. 
